# The Short-Term Cost-Effectiveness of Once-Daily Liraglutide Versus Once-Weekly Exenatide for the Treatment of Type 2 Diabetes Mellitus in the United States

**DOI:** 10.1371/journal.pone.0121915

**Published:** 2015-04-07

**Authors:** Bruce Wang, Joshua A. Roth, Hiep Nguyen, Eugene Felber, Wes Furnback, Louis P. Garrison

**Affiliations:** 1 Elysia Group, LLC, New York, New York, United States of America; 2 Department of Pharmacy, University of Washington, Seattle, Washington, United States of America; 3 Public Health Sciences Division, Fred Hutchinson Cancer Research Center, Seattle, Washington, United States of America; 4 AstraZeneca, Fort Washington, Pennsylvania, United States of America; 5 Bristol-Myers Squibb Company, New York, New York, United States of America; University of Lancaster, UNITED KINGDOM

## Abstract

**Background:**

Type 2 diabetes mellitus (T2DM) is a chronic metabolic disease with substantial morbidity, mortality, and economic impacts. Glucagon-like peptide-1 (GLP-1) receptor agonists, such as once-daily (QD) liraglutide and once-weekly (QW) exenatide, are FDA-approved treatment for T2DM. Head-to-head trials and meta-analyses comparing these agents have reported clinically meaningful improvements but small differences in glycemic control between both agents. In this study, we calculate and compare the cost-effectiveness implications of these alternative effectiveness outcomes.

**Methods:**

We developed a decision model to evaluate the short-term cost-effectiveness of exenatide QW 2 mg versus liraglutide QD 1.8 mg in T2DM patients, with effectiveness measured as reduction in glycated hemoglobin (HbA1c). In the base case, the model tracks change in HbA1c and direct medical expenditure over a 6-month time horizon. We calculated and compared the cost per 1% reduction in HbA1c of models populated with clinical data from a head-to-head randomized, controlled trial (DURATION-6) and a network meta-analysis. Expenditure inputs were derived from wholesale acquisition costs and published sources.

**Results:**

In the base case, 6-month expenditure for the liraglutide and exenatide strategies were $3,509 and $2,618, respectively. Using clinical data from DURATION-6 and the network meta-analysis, the liraglutide strategy had an incremental cost per 1% reduction in HbA1c of $4,773 and $27,179, respectively. The most influential model parameters were drug costs, magnitude of HbA1c reduction in patients on treatment for >1 month, and liraglutide gastrointestinal adverse event rate. In probabilistic sensitivity analyses (PSA) using DURATION-6 data, the exenatide strategy was optimal at willingness-to-pay levels below $4,800 per 1% reduction in HbA1c. In a PSA using meta-analysis data, the exenatide strategy was dominant.

**Conclusions:**

Our modeled results demonstrate that the effectiveness and cost-effectiveness of liraglutide QD 1.8 mg relative to exenatide QW 2 mg depend largely on the chosen source of the clinical data.

## Introduction

Type 2 diabetes mellitus (T2DM) is a chronic metabolic disease that affects approximately 23 million Americans [1]. By 2030, it is estimated that as many as 44 million Americans will be diagnosed with T2DM [2,3]. Given the substantial prevalence of T2DM, and the noted morbidity, mortality, and economic impacts of the disease, the identification of cost-effective treatment strategies is of high public health and economic importance [2–4].

Glycated hemoglobin (HbA1c) reflects average levels of blood glucose and is a frequently used measure of T2DM treatment efficacy and effectiveness [5]. This outcome is important, because every one-percentage point reduction in HbA1c is associated with a 40% reduction in the risk of microvascular disease complications [6]. To achieve HbA1c reduction, guidelines recommend lifestyle modification (i.e., exercise, diet, weight loss) as a first-line treatment, and the addition of metformin therapy for patients who fail to improve hyperglycemia with lifestyle modification alone [7]. However, as many as 50% of patients with T2DM remain hyperglycemic after lifestyle modification and pharmacologic monotherapy interventions, and for those patients, guidelines recommend the addition of a second agent, such as a glucagon-like peptide-1 (GLP-1) receptor agonist [8].

A growing body of evidence suggests that the GLP-1 receptor agonists once-weekly (QW) exenatide 2 mg and once-daily (QD) liraglutide 1.8 mg are efficacious at reducing HbA1c levels in patients with otherwise uncontrolled T2DM [9,10]. However, the relative effectiveness and cost-effectiveness of these agents remain uncertain due to the small and variable differences that have been demonstrated in prior studies [9,10]. The DURATION-6 open-label, randomized, controlled trial compared the 6-month head-to-head outcomes of liraglutide QD 1.8 mg and exenatide QW 2 mg added to lifestyle modification and metformin therapy in 911 adult patients with T2DM [9]. In that trial, the liraglutide arm had slightly greater HbA1c reduction relative to the exenatide arm (–0.21%, 95% CI: –0.08 to—0.33), but also had more adverse events (e.g., nausea, vomiting, diarrhea) [9]. In another recent study, Scott and colleagues conducted a network meta-analysis that pooled outcomes from 22 clinical studies (11,049 total patients) and demonstrated no meaningful differences in HbA1c reduction between liraglutide QD 1.8 mg and exenatide QW 2 mg (–0.03%, 95% CI: –0.14 to 0.18) [10]. These findings demonstrate the need for additional evidence to help patients, clinicians, and payers better understand benefit-risk and economic tradeoffs.

The objective of this analysis was to evaluate the short-term cost-effectiveness of liraglutide QD 1.8 mg versus exenatide QW 2 mg in adult T2DM. Our findings provide needed evidence about the value of the small differences in reported effectiveness of these agents and can inform the design of future GLP-1 receptor agonist comparative effectiveness studies in T2DM.

## Materials and Methods

### Overview

**Fig 1:**
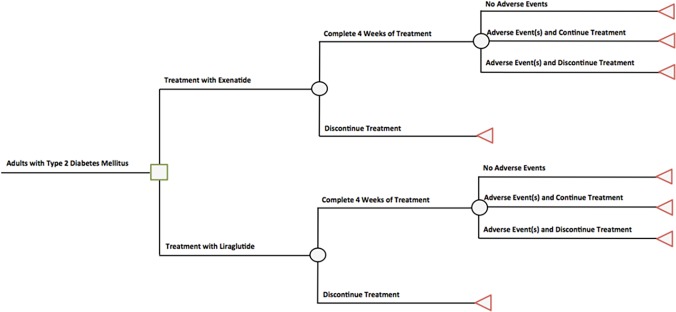
Simulation model decision tree. The patient cohort receives exenatide or liraglutide, and moves through the tree from left to right.

We developed a simulation model in Microsoft Excel (Microsoft Inc., Redmond, WA) to estimate 6- and 12-month health outcomes for a cohort of adult patients with T2DM who had not achieved glycemic control after treatment with lifestyle modification and metformin therapy. The model compared health outcomes for the cohort in two GLP-1 receptor agonist treatment strategies: 1) exenatide QW 2 mg added to lifestyle modification and metformin therapy, and 2) liraglutide QD 1.8 mg added to lifestyle modification and metformin therapy (Fig 1). The cohort was tracked for adverse events, treatment discontinuation, HbA1c reduction, and associated direct medical expenditure (hereafter “costs”) from a payer perspective. Two clinical data sources were considered: 1) the DURATION-6 phase III randomized, controlled trial (NCT01029886), and 2) Scott and colleagues’ network meta-analysis [9,10]. Direct costs and HbA1c changes were tracked over 6-month and 1-year time horizons in accordance with the reported interests of United States (US) health insurers, and as in prior studies [11–14].

### Model Structure

The simulation model was developed as a decision tree (Fig 1) that tracks key events in the short-term treatment of T2DM with GLP-1 receptor agonists added to lifestyle modification and metformin therapy. The tracked events include adverse events (trial-based), treatment discontinuation at 1 month, and treatment discontinuation at 3 months. These key events were selected based on discussions with clinical experts in T2DM care.

### Model Inputs

We derived model inputs from the DURATION-6 trial, Scott and colleagues’ meta-analysis, other peer-reviewed literature sources, and wholesale acquisition costs (WACs), as explained below.

#### GLP-1 Receptor Agonist Use and Effectiveness Inputs

GLP-1 receptor agonist use and effectiveness inputs were derived from the DURATION-6 trial and Scott and colleagues’ network meta-analysis (Table 1). In DURATION-6, the 6-month mean reduction in HbA1c was greater in the liraglutide arm relative to the exenatide arm (–1.48% vs. –1.28%) [9]. In Scott and colleagues’ network meta-analysis, the mean reduction in HbA1c was also greater with liraglutide relative to exenatide QW, but the difference was much smaller relative to the DURATION-6 trial (–1.18% vs. –1.15%) [10]. The decision model applied these estimates of HbA1c reduction over 6-month and 1-year time horizons, assuming equivalent HbA1c reduction in patients who received the full duration of treatment over both time horizons.

**Table 1:** Simulation model inputs.

**Model Input**	**Point Estimate**	**Low**	**High**	**Data Source**	**Distribution**
**Exenatide 2 mg**					
** Effectiveness**					
** **DURATION-6 Trial Reduction in HbA_1c_	1.28%	1.18%	1.38%	[9]	Beta
** **Meta-Analysis Reduction in HbA_1c_	1.15%	1.00%	1.31%	[10]	Beta
** Discontinuation Rate**					
** **Before Week 4	0%	0%	1%	[9]	Beta
** **After Week 4	13.23%	10.59%	15.88%	[9]	Beta
** Drug Cost (2014 $USD)**					
** **Monthly cost of exenatide 2 mg (4 Pack)	$407	$367	$448	WAC	Normal
** Adverse Event Rates**					
** **Patients Experiencing 1+ Adverse Events	61.39%	49.11%	73.67%	[9]	Beta
** **Gastrointestinal Disorders	39.93%	31.94%	47.92%	[9]	Beta
**Liraglutide 1.8 mg**					
** Effectiveness**					
** **DURATION-6 Trial Reduction in HbA_1c_	1.48%	1.38%	1.58%	[9]	Beta
** **Meta-Analysis Reduction in HbA_1c_	1.18%	1.04%	1.32%	[10]	
** Discontinuation**					
** **Before Week 4	3.33%	2.66%	4.00%	[9]	Beta
** **After Week 4	9.78%	7.82%	11.74%	[9]	Beta
** Drug Cost (2014 $USD)**					
** **Monthly cost of liraglutide 1.8 mg (30 Pack)	$536	$482	$589	WAC	Normal
** **Needle cost	$9	$8	$10	Assumption	Normal
** Adverse Event Rates**					
** **Patients Experiencing 1+ Adverse Events	64.89%	51.91%	77.87%	[9]	Beta
** **Gastrointestinal Disorders	54.7%	43.78%	65.66%	[9]	Beta
**Other Costs**					
** Adverse Event Costs (2014 $USD)**					
** **Gastrointestinal Disorders	$1,113	$890	$1,335	Expert Opinion	Normal

HbA1c = glycated hemoglobin; $USD = United States dollars; WAC = wholesale acquisition cost

#### Adverse Events

The simulation model also incorporated liraglutide and exenatide treatment-emergent adverse events (TEAEs) from the DURATION-6 trial. We focused on gastrointestinal events, the only type of event that differed significantly between the liraglutide and exenatide arms [9]. Specifically, we included nausea, diarrhea, vomiting, constipation, abdominal pain, and dyspepsia, which were the gastrointestinal events present in at least 5% of either treatment arm. For the liraglutide arm, 64.89% of patients who remained after week 4 experienced ≥1 TEAE; of these patients, 54.72% experienced a gastrointestinal adverse event. In the exenatide arm, 61.39% of all patients experienced ≥1 TEAE; of these patients, 39.93% experienced a gastrointestinal adverse event.

#### Treatment Discontinuation Inputs

We modeled treatment discontinuation based on patterns from the DURATION-6 trial. In that study, no patients in the exenatide arm discontinued in the first month of treatment, and 3.33% of patients in the liraglutide arm discontinued in the first month of treatment [9]. After the first month of treatment, 13.23% of the patients in the exenatide arm and 9.78% of the patients in the liraglutide arm discontinued therapy [9].

Among patients who discontinued treatment, we assumed that those stopping treatment in the initial 4 weeks incurred drug cost for 28 days and that those who discontinued after 4 weeks incurred drug cost for 12 weeks of treatment. Additionally, discontinuation was modeled to impact treatment effectiveness, with the assumption that patients discontinuing therapy returned to baseline HbA1c level.

#### Treatment Adherence

Given the lack of evidence about adherence and HbA1c reduction with GLP-1 receptor agonists, we assumed 100% adherence to the study medications in both treatment strategies.

#### Cost Inputs

Patients incurred treatment-related costs based on the economic inputs displayed in Table 1. The base-case monthly cost of treatment with liraglutide QD 1.8 mg and exenatide QW 2 mg was derived from the 2014 WACs. In the base case, we assumed that there was no cost offset for patient copayment or health plan discounts (i.e., rebates).

The costs of treating adverse events were obtained from the published literature and informed by clinical experts. Patients who experienced an adverse event were assigned a probability of having an emergency department, inpatient, or outpatient visit, and weighted average event cost was calculated and applied accordingly in 2014 US dollars adjusting for inflation using the CPI.

We did not explicitly model lifestyle modification or metformin therapy costs, as they are involved in both strategies under comparison, and therefore have no or little impact on incremental outcomes.

We assumed that treatment discontinuation following adverse events did not involve any additional costs.

#### Model Outcomes

The primary outcome of our analysis was the incremental cost per 1% HbA1c reduction. We calculated the incremental cost-effectiveness ratio (ICER) by dividing the difference in total cost for liraglutide versus exenatide by the difference in HbA1c reduction for liraglutide versus exenatide. We chose HbA1c reduction as our measure of effectiveness because it is a standard T2DM outcome that has clinical and economic significance for patients, clinicians, and payers. Evaluating this outcome over 6-month and 1-year time horizons is consistent with a number of prior analyses of GLP-1 receptor agonists in T2DM, and corresponds to the reported preference of many US stakeholders to consider cost-effectiveness based on clinical outcome measures (rather than quality-adjusted life-years) over short time horizons [11–14].

#### Sensitivity Analyses

We evaluated outcome uncertainty using one-way and probabilistic sensitivity analyses. In the one-way sensitivity analysis, low- and high-input value estimates were propagated through the model, and we obtained the resulting range of incremental HbA1c reduction and costs for each individual input. The one-way sensitivity analysis results are presented in the form of tornado diagrams displaying the 10 most influential model inputs. We also conducted a probabilistic sensitivity analysis using Monte Carlo simulation [15–17]. This approach involved specifying the distribution of model inputs (costs = normal, probabilities = beta), simultaneously sampling parameter sets from the distributions, and propagating the values through the model framework to calculate the joint distribution of model outcomes [15,16]. The probabilistic sensitivity analysis results were used to calculate 95% credible intervals (95% CIs) around model outcomes, and we display these results in the form of cost-effectiveness acceptability curves [18].

#### Willingness to Pay Per 1% HbA1c Reduction

There is not an explicit willingness-to-pay threshold for HbA1c reduction in the US. Accordingly, we evaluated cost-effectiveness over a plausible range of willingness-to-pay thresholds from $500 to $5,000 per 1% HbA1c reduction.

## Results

### Base-Case Results (6-Month Time Horizon)

The outcomes of the 6-month horizon analysis from DURATION-6 and Scott and colleagues’ meta-analysis clinical data sources are shown in Tables 2 and 3, respectively. Compared with the exenatide strategy, the liraglutide strategy was more costly based on the DURATION-6 trial (incremental HbA1c reduction = 0.19%, incremental cost = $891) and Scott and colleagues’ meta-analysis (incremental HbA1c reduction = 0.03%, incremental cost = $891). Accordingly, the 6-month cost per 1% reduction in HbA1c for liraglutide versus exenatide was $4,773 based on the DURATION-6 trial, and $27,179 based on Scott and colleagues’ meta-analysis.

**Table 2:** Base-case model results using DURATION-6 trial clinical inputs.

**Strategy**	**Drug Cost**	**Adverse Event Cost**	**Discontinuation Cost**	**Total Cost**	**HbA1c Reduction**	**ICER**
Liraglutide QD 1.8 mg	$3,077	$432	$0	$3,509	–1.54%	-
Exenatide QW 2 mg	$2,345	$273	$0	$2,618	–1.36%	-
Difference	$732	$159	$0	$891	0.19%	$4,773

HbA1c = glycated hemoglobin; ICER = incremental cost-effectiveness ratio; QD = once daily; QW = once weekly

The liraglutide incremental cost-effectiveness ratio (ICER) indicates that strategy is expected to cost $4,773 for a 1% reduction in HbA1c relative to the exenatide strategy.

**Table 3:** Base-case model results using Scott and colleagues’ meta-analysis clinical inputs.

**Strategy**	**Drug Cost**	**Adverse Event Cost**	**Discontinuation Cost**	**Total Cost**	**HbA1c Reduction**	**ICER**
Liraglutide QD 1.8 mg	$3,077	$432	$0	$3,509	-1.24%	-
Exenatide QW 2 mg	$2,345	$273	$0	$2,618	-1.21%	-
Difference	$732	$159	$0	$891	0.03%	$27,179

HbA1c = glycated hemoglobin; ICER = incremental cost-effectiveness ratio; QD = once daily; QW = once weekly

The liraglutide incremental cost-effectiveness ratio (ICER) indicates that strategy is expected to cost $27,179 for a 1% reduction in HbA1c relative to the exenatide strategy.

### 1-Year Time Horizon Results

Over a 1-year time horizon, compared with the exenatide strategy, the liraglutide strategy was again more costly based on the DURATION-6 trial (incremental HbA1c = 0.19%, incremental cost = $1,604) and Scott and colleagues’ meta-analysis (incremental HbA1c reduction = 0.03%, incremental cost = $1,604). Accordingly, the 1-year cost per 1% reduction in HbA1c for liraglutide versus exenatide was $8,589 based on the DURATION-6 trial, and $48,912 based on Scott and colleagues’ meta-analysis.

### Sensitivity Analysis Results

**Fig 2:**
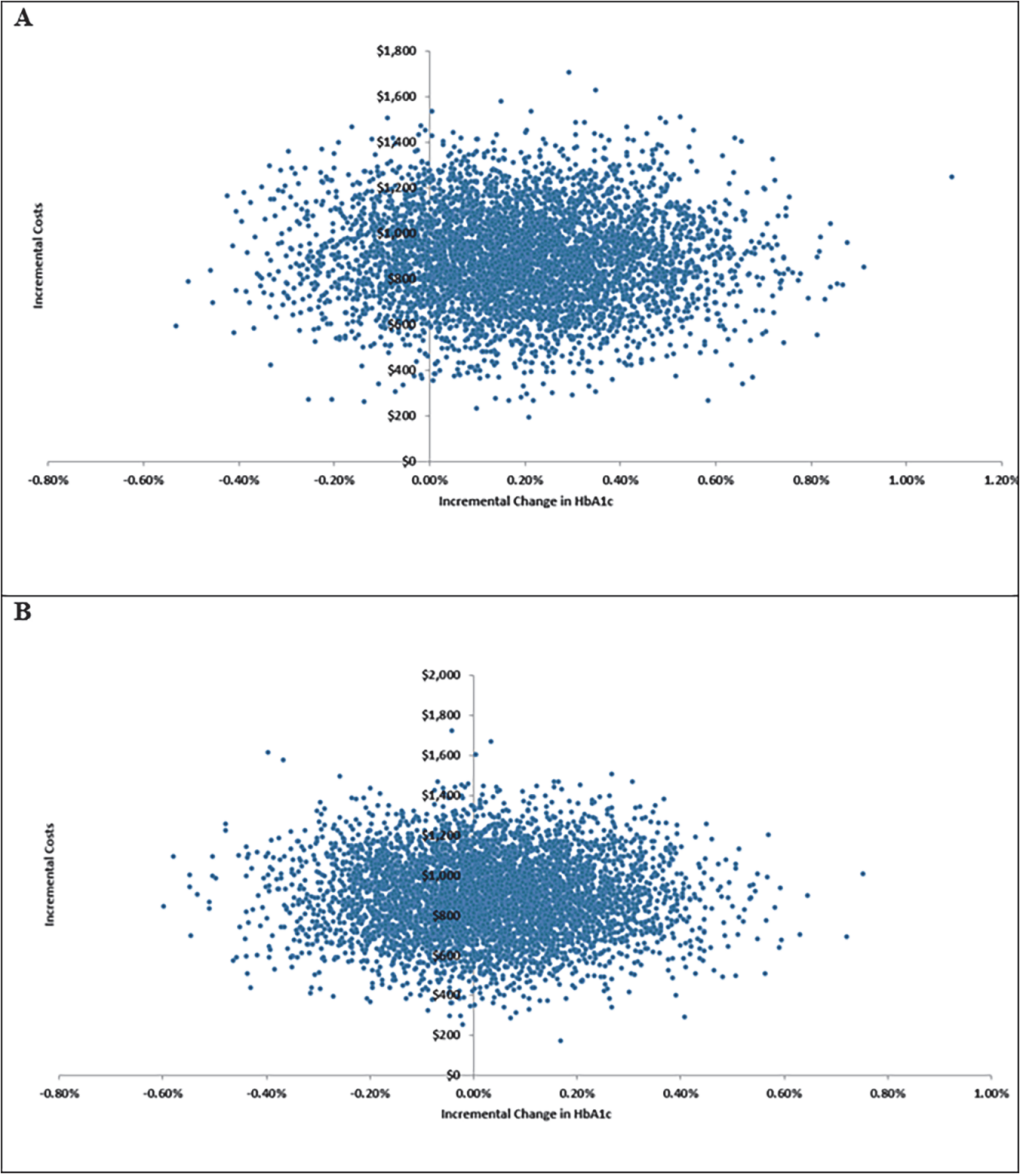
Cost-effectiveness plane scatter plot for the (A) DURATION-6 clinical input and (B) Scott and colleagues’ meta-analysis clinical input analyses. The figure displays the incremental HbA1c (x axis) and incremental cost (y axis) outcomes of 5,000 Monte Carlo simulations. Positive HbA1c values reflect greater effectiveness of liraglutide, and negative values reflect greater effectiveness of exenatide. Positive cost values reflect higher cost for liraglutide, and negative cost values reflect higher cost for exenatide.

Fig 2A shows the incremental HbA1c reduction and cost outcomes for 5,000 Monte Carlo simulation runs based on clinical data from the DURATION-6 trial. In those simulations, the liraglutide strategy resulted in greater HbA1c reduction in 81% of runs and greater cost in 100% of runs. Fig 2B shows similar outcomes based on Scott and colleagues’ meta-analysis. Using that clinical data source, the liraglutide strategy resulted in greater HbA1c reduction in 58% of runs and greater cost in 100% of runs.

**Fig 3:**
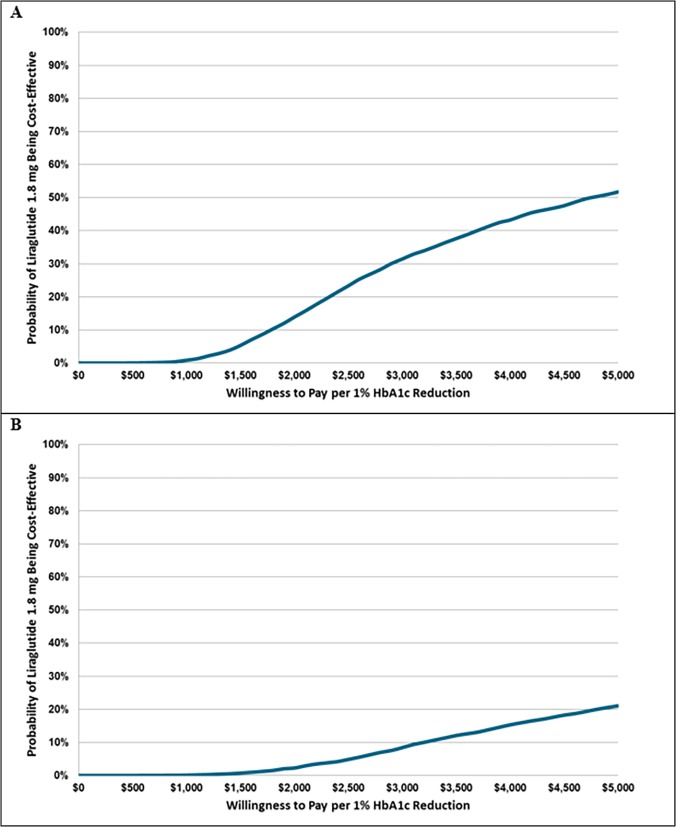
Cost-effectiveness acceptability curve for the (A) DURATION-6 clinical input and (B) Scott and colleagues’ meta-analysis clinical input analyses. The figure displays the probability that the liraglutide strategy is a cost-effective alternative to the exenatide strategy over a plausible range of willingness to pay per 1% HbA1c reduction.

Fig 3A displays cost-effectiveness acceptability curves based on the DURATION-6 trial and Scott and colleagues’ meta-analysis for the base-case 6-month analysis. Using DURATION-6 trial clinical data, the liraglutide strategy is expected to be cost-effective at a willingness to pay per 1% HbA1c reduction of approximately $4,800 or greater. This indicates that if willingness to pay per 1% HbA1c reduction is less than $4,800, exenatide is considered the optimal treatment strategy. Using Scott and colleagues’ meta-analysis clinical data, the exenatide strategy is expected to be the optimal strategy across the entire range of willingness to pay evaluated in this analysis (i.e., up to $5,000 per 1% HbA1c reduction).

**Fig 4:**
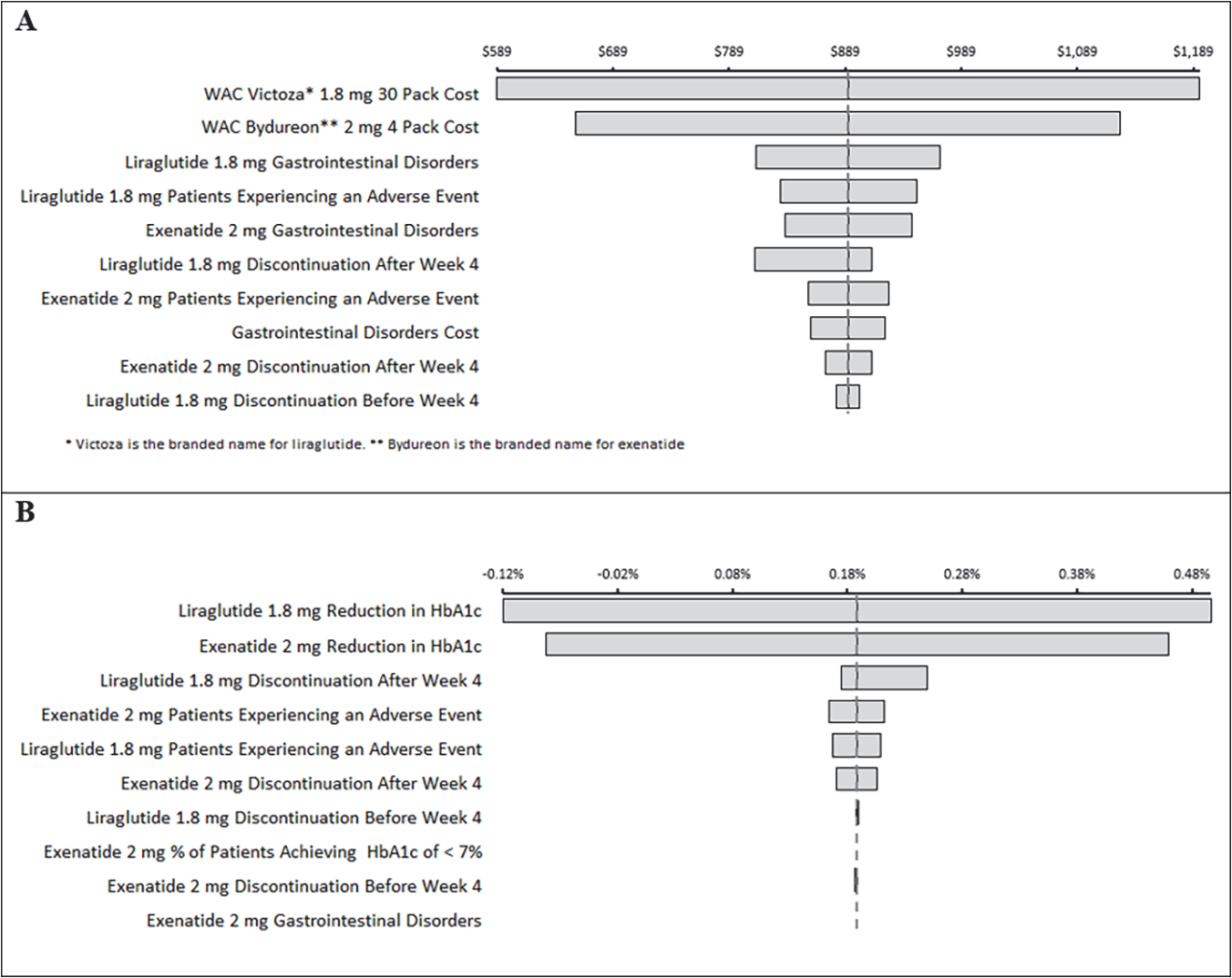
One-way sensitivity analysis tornado diagrams. The figure displays the most influential model inputs for (A) incremental cost and (B) incremental HbA1c reduction outcomes in the DURATION-6 clinical input analysis.

In a one-way sensitivity analysis examining incremental cost, the cost of liraglutide and exenatide, the liraglutide and exenatide adverse event rates, and the cost of treating gastrointestinal adverse events were most influential (Fig 4A). In a one-way sensitivity analysis of incremental HbA1c reduction, the most influential parameters were the effectiveness of liraglutide and exenatide in patients who completed the full treatment duration, the exenatide discontinuation rate before week 4 of treatment, and the liraglutide discontinuation rate after week 4 of treatment (Fig 4B).

## Discussion

We conducted an evaluation of the short-term cost-effectiveness of the GLP-1 receptor agonists liraglutide and exenatide, with cost-effectiveness assessed as cost per 1% reduction in HbA1c. This measure is a common T2DM outcome and has well-established implications for microvascular disease risk and associated costs. Given inconsistent findings about the relative effectiveness of liraglutide and exenatide in prior studies, we evaluated results with two different clinical data sources: 1) the DURATION-6 randomized, controlled trial, and 2) a network meta-analysis conducted by Scott and colleagues [9,10]. We found that the short-term incremental cost per 1% HbA1c reduction estimates varied substantially between analyses, with the DURATION-6–based analysis resulting in a liraglutide QD 1.8 mg ICER of $4,773, and the Scott and colleagues’ meta-analysis resulting in a liraglutide QD 1.8 mg ICER of $27,179.

This study has important implications for stakeholders considering the relative value of liraglutide and exenatide. Previous studies have demonstrated a small difference in short-term efficacy between these agents, with liraglutide resulting in a 0.03 to 0.19 greater reduction in HbA1c [9,10]. Although the effectiveness data for this model were obtained from the two sources that directly and indirectly compared liraglutide and exenatide, these differences should be taken in the context of the overall clinical evidence for the two GLP-1 receptor agonists. In the network meta-analysis by Scott and colleagues, the 0.03 difference in HbA1c was not statistically significant, suggesting similar effectiveness for liraglutide and exenatide QW when the evidence is pooled across 22 studies. The similar effectiveness may be partially explained by the overlap in the range of absolute reduction in HbA1c for the two GLP-1 receptor agonists: –1.3% to—1.9% for exenatide QW and—1.0 to—1.5% for liraglutide [9,19–29].

The cost estimates from our analysis suggest higher treatment-related costs for liraglutide compared with exenatide QW. Although we included only pharmacy and costs associated with TEAEs, our results are consistent with previous studies that have also demonstrated higher 6-month direct medical expenditure of approximately $200 to $700 with liraglutide versus exenatide QW [12,13]. In this study, we synthesized this effectiveness and expenditure evidence and found that liraglutide is expected to cost between $4,800 and $27,200 per 1% reduction in HbA1c. Although there is not a well-established willingness to pay per 1% HbA1c reduction, we believe it is reasonable to evaluate an implied threshold based on the results of prior studies that have evaluated the cost consequences of reducing HbA1c [14,30,31]. In this context, our findings suggest that liraglutide has poor value relative to exenatide QW based on the fact that prior studies demonstrate reduced annual expenditure of approximately $300 in patients with good versus poor HbA1c control, and even in our most favorable scenario, the liraglutide cost per 1% reduction in HbA1c is much greater [6].

Our findings also have important implications for the design of future studies evaluating the comparative effectiveness of liraglutide and exenatide for the treatment of T2DM. There is currently limited real-world evidence available to inform stakeholder evaluation of the benefit-risk tradeoffs and cost-effectiveness of these agents. Future studies should compare effectiveness, adverse event rates, adherence rates, and expenditure in patients being treated in community settings to provide information about health outcomes in typical patients with T2DM. Additionally, given the common use of HbA1c reduction as an end point in clinical trials, observational studies, and simulation modeling analyses, future research should investigate the clinical and economic impacts of key HbA1c reduction thresholds (with particular attention the common end point of 1% HbA1c reduction).

This study has several limitations. First, we restricted our comparison to exenatide QW versus liraglutide QD 1.8 mg. It should be noted that liraglutide QD is also available in a 1.2-mg formulation, which has different effectiveness, adverse event, and cost implications. However, evidence from randomized trials and observational studies suggests that liraglutide 1.2 mg is significantly less effective than exenatide QW [10,32]. Accordingly, we compared with liraglutide 1.8 mg because it has the most similar effectiveness and is the most clinically relevant comparator for exenatide QW. Second, we restricted the costs in the analysis to drugs and major TEAEs, as they were expected to be the major drivers of the relative cost-effectiveness of exenatide QW and liraglutide. Therefore, the model did not account for the cost of all possible adverse events or the cost of switching to other therapies. Third, the analysis evaluates cost-effectiveness as cost per 1% HbA1c reduction. Given limited evidence about the cost impacts of reducing HbA1c, it can be difficult to establish a precise willingness-to-pay threshold for this clinical outcome. Fourth, the analysis takes a payer perspective over a short-term time horizon. Alternative perspectives and time horizons may result in different cost-effectiveness implications. Fifth, the choice of WAC prices excludes potential discounts or rebates, which payers may need to consider in their decision-making processes. The sensitivity analysis suggests the costs of the drugs influence the findings. Lastly, this analysis does not consider any potential benefits of weekly injection (with exenatide QW) relative to daily injection (with liraglutide). These different schedules may create additional value for patients, payers, and other stakeholders.

## Conclusion

This is the first short-term evaluation of cost-effectiveness of exenatide QW versus liraglutide in T2DM. Our modeled results demonstrate that the effectiveness and cost-effectiveness of liraglutide QD 1.8 mg relative to exenatide QW 2 mg depend largely on the chosen source of the clinical data. Thus, our findings suggest that in a US setting, the cost-effectiveness of liraglutide QD 1.8 mg relative to exenatide QW 2 mg is highly uncertain. Future studies should evaluate the comparative effectiveness of these GLP-1 receptor agonist strategies in community settings to facilitate more precise estimation of clinical and economic tradeoffs.

## Supporting Information

S1 Table Adverse event costs. The table shows how the adverse event cost was calculated.
(DOCX)
