# Supplementary material for: The Short-Term Cost-Effectiveness of Once-Daily Liraglutide Versus Once-Weekly Exenatide for the Treatment of Type 2 Diabetes Mellitus in the United States
Source: PLoS One. 2015 Apr 7;10(4):e0121915. doi: 10.1371/journal.pone.0121915 (PMC4388383; doi:10.1371/journal.pone.0121915)
Supplement: S1 Table — The table shows how the adverse event cost was calculated. (DOCX) [file pone.0121915.s001.docx]

| **Event** | **Number of Events [9]** | **Weight** | **Cost** | **Weighted Cost** |
| --- | --- | --- | --- | --- |
| Nausea | 136 | 36.66% | $1,170 | $428.93 |
| Diarrhea | 89 | 23.99% | $566 | $135.73 |
| Vomiting | 65 | 17.52% | $2,153 | $377.18 |
| Constipation | 43 | 11.59% | $305 | $35.34 |
| Dyspepsia | 38 | 10.24% | $1,324 | $135.62 |
| ***Total*** | ***371*** | ***100.00%*** |  | ***$1,112.80*** |
| *Costs were calculated using the MEPS costs and expert opinion to find the probability of an emergency department, inpatient, or outpatient visit for each adverse event. Expert opinion was also used to provide the average drug cost to treat each adverse event. | | | | |
|  |  |  |  |  |

**REFERENCES**

Agency for Healthcare Research & Quality. Medical expenditures panel survey: table 1: total utilization and mean expenses per visit by type of ambulatory health care service, 2010. http://meps.ahrq.gov/mepsweb/data_stats/summ_tables/hc/mean_expend/2010/table1.pdf Accessed October 8, 2014

Agency for Healthcare Research & Quality. Medical expenditures panel survey: table 2: total utilization and mean expenses per visit by type of ambulatory health care service, 2010. http://meps.ahrq.gov/mepsweb/data_stats/summ_tables/hc/mean_expend/2010/table2.pdf Accessed October 8, 2014
